# Reducing spillage during emergency stoma formation in patients with obstructed and unprepared bowel

**DOI:** 10.1308/003588413X13511609958055c

**Published:** 2013-03

**Authors:** S Pericleous, JA Hannay, T Sharma, A Macdonald

**Affiliations:** NHS Lanarkshire, UK

## Background

Stoma formation is often complicated by early skin irritation and infection. Rates for local irritation are reported at 3–42% while peristomal infections and abscesses occur in 2–14.8% of cases. Intraoperative bowel content spillage is an avoidable risk factor.^1^ We describe a cost efficient method of minimising spillage and forcepselated bowel trauma using a Foley catheter. This technique is particularly suited when performing a trephine stoma on unprepared bowel in the emergency setting.

**Figure 1:**
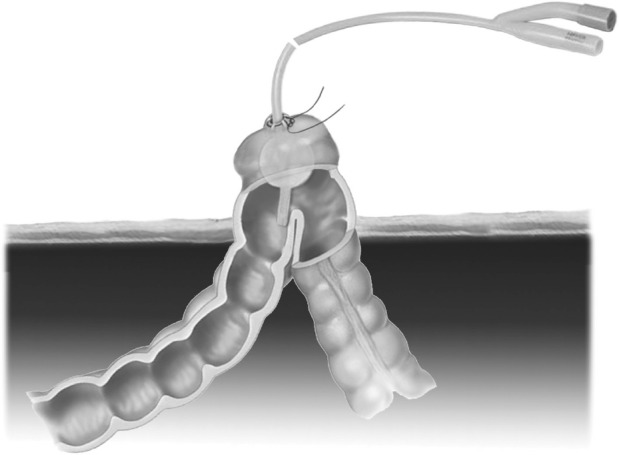
Anatomical representation of the technique, showing the Foley catheter with an inflated 10ml balloon secured in place with a purse string suture

## Technique

Candidate stoma sites are marked preoperatively. We use computed tomography to identify a suitable section of bowel. A trephine is performed through the abdominal wall in the standard fashion.^2^ A 3/0 monofilament purse string suture is then placed in the antimesenteric border of the dilated bowel and a stab incision is made at the centre. A large bore Foley catheter is inserted immediately and the balloon inflated with 10ml of water. Gentle traction is applied to the catheter and the purse string suture is tightened to ensure a competent seal. Dilated bowel can then be decompressed by attaching a suction device to the catheter. Luminal lavage may subsequently be performed in a controlled manner via the catheter by alternating instillation of warmed saline with suction. The resultant flaccid bowel loop is delivered atraumatically for stoma formation by traction on the Foley catheter.

## Discussion

Our technique is simple, safe, inexpensive and uses equipment readily available in most hospitals. It allows the surgeon to perform a controlled bowel decompression and irrigation washout without additional incisions or trauma.
